# Diagnostic Performance of Initial Serum Albumin Level for Predicting In-Hospital Mortality among Necrotizing Fasciitis Patients

**DOI:** 10.3390/jcm7110435

**Published:** 2018-11-10

**Authors:** Chia-Peng Chang, Wen-Chih Fann, Shu-Ruei Wu, Chun-Nan Lin, I-Chuan Chen, Cheng-Ting Hsiao

**Affiliations:** 1 Department of Emergency Medicine, Chang Gung Memorial Hospital, No.6, Sec. W., Jiapu Rd., Puzi City, Chiayi County 613, Taiwan; giovanni850730@yahoo.com.tw (C.-P.C.); patrickfann@cgmh.org.tw (W.-C.F.); p5832@cgmh.org.tw (C.-N.L.); giomacky@gmail.com (I.-C.C.); 2 Department of Medicine, Chang Gung University, Taoyuan 333, Taiwan; 3 Department of Pediatrics, Kaohsiung Veterans General Hospital, Kaohsiung 813, Taiwan; sierrawu@gmail.com

**Keywords:** albumin, mortality, necrotizing fasciitis

## Abstract

*Background*: Hypoalbuminemia is known to be associated with adverse outcomes in critical illness. In this study, we attempted to identify whether hypoalbuminemia on emergency department (ED) arrival is a reliable predictor for in-hospital mortality in necrotizing fasciitis (NF). patients. *Method*: A retrospective cohort study of hospitalized adult patients with NF was conducted in a tertiary teaching hospital in Taiwan between March 2010 and March 2018. Blood samples were collected in the ED upon arrival, and serum albumin levels were determined. We evaluated the predictive value of serum albumin level at ED presentation for in-hospital mortality. All collected data were statistically analyzed. *Result*: Of the 707 NF patients, 40 (5.66%) died in the hospital. The mean serum albumin level was 3.1 ± 0.9 g/dL and serum albumin levels were significantly lower in the non-survivor group than in the survivor group (2.8 ± 0.7 g/dL vs. 3.5 ± 0.8 g/dL). In the multivariable logistic regression model, albumin was significantly associated with in-hospital mortality (odds ratio (OR) 0.92, 95% confidence interval (CI) 0.88–0.96, *p* < 0.001). The area under-the-receiver-operating-characteristic curve (AUC) for in-hospital survival was 0.77 (95% CI 0.72–0.82) and corresponding sensitivity, specificity, positive predictive value, negative predictive value, and positive and negative likelihood ratio were 66%, 74%, 33%, 88%, 2.25, and 0.48, respectively. High sensitivity (96%) for survival was shown at albumin level of 4.0 g/dL and high specificity (91%) for mortality was shown at a level of 2.5 g/dL. *Conclusion*: Initial serum albumin levels strongly predicted in-hospital mortality among patients with necrotizing fasciitis. NF patients with hypoalbuminemia on ED arrival should be closely monitored for signs of deterioration and early and aggressive intervention should be considered to prevent mortality.

## 1. Introduction

Necrotizing fasciitis (NF) is a serious form of infection involving rapidly spreading inflammation and extensive necrosis of the skin, subcutaneous tissue, and superficial fascia [1]. The treatment of choice for NF is rapid surgical debridement and broad-spectrum antibiotic therapy [2]. Even with aggressive treatment, patients may suffer mortality and significant morbidity such as amputation and organ failure [3,4,5].

Various factors have been shown to be associated with mortality among patients with necrotizing fasciitis. Most of the identified factors have been patient characteristics such as older age, liver cirrhosis, cancer, and peripheral vascular disease. Among widely used laboratory markers, band polymorphonuclear neutrophils >10%, serum creatinine level >2 mg/dL, hyperlactatemia, laboratory risk indicator of necrotizing fasciitis (LRINEC) score >8, and serum albumin levels have been identified in association with mortality [6,7,8]. Serum albumin levels may also serve as a prognostic factor for critical patients, and the diagnostic performance of this parameter in critical care is well-known [9,10,11]. However, literature about the diagnostic performance of serum albumin levels among patients with necrotizing fasciitis is limited.

In the present study, we determined the association between in-hospital mortality and serum albumin levels at presentation to the emergency department (ED) among patients with necrotizing fasciitis. Additionally, the diagnostic performance of serum albumin levels was investigated.

## 2. Material and Methods

### 2.1. Patient Selection

The institutional review board of our hospital approved this retrospective study. In all, 707 patients were enrolled based on two criteria: (1) surgically proven diagnosis of NF and (2) treatment received between March 2010 and March 2018 in our hospital. All patients were assessed by emergency physicians as soon as they were admitted. They received broad-spectrum antibiotic treatment for anaerobic and aerobic bacteria, as well as early surgical debridement including fasciotomy or primary amputation post-diagnosis. Each patient’s medical record was screened for documentation of NF to confirm the diagnosis. Blood samples were collected in the ED upon arrival, and the serum albumin levels were determined. Baseline demographic characteristics, laboratory findings, serum albumin, and clinical presentation were compared between survivors and non-survivors groups.

### 2.2. Data Analysis

We reviewed charts and recorded variables including age, systolic and diastolic blood pressure at triage, comorbidities, discharge diagnosis, and mortality or survival on discharge. Patients with in-hospital mortality was defined as a death occurring in the hospital after admission, also known as the ‘non-survivors group’, or otherwise as the ‘survivors group’. We defined these variables as follows: episodes of hypotension, episodes of systolic blood pressure less than 90 mmHg at the ED; hypothermia, body temperature less than 36 degrees Celcius at the ED; hyperthermia, body temperature greater than or equal to 38 degrees Celcius at the ED ; acidosis, pH less than 7.35 in arterial blood gas test at the ED; coagulopathy, a prolonged prothrombin time test (international normalized ratio) result greater than 1.5 at the ED; thrombocytopenia, platelet counts less than 100 × 10^3^ uL at the ED; anemia, hemoglobin less than 10 mg/dL at the ED; hypoxia, episodes of oxygen saturation less than 90% at the ED. Prothrombin time test, hemoglobin, platelet counts, blood gas test, serum lactate, albumin, sodium, creatinine, and CRP (C-reactive protein) were assessed by first laboratory analyses in the ED.

Statistical analyses were conducted using SPSS 20.0. Assumptions of normality and homogeneity of variance were first checked. For continuous variables with a skewed distribution, descriptive results were expressed as medians and interquartile ranges. The Mann–Whitney U test was used to determine the differences between two groups, and the Kruskal–Wallis H test was used to analyze the differences among groups. Univariate binary and multivariate logistic regression analyses were performed to investigate whether serum albumin remained to be most significantly associated with in-hospital mortality. The model fit was assessed with the Hosmer–Lemeshow goodness-of-fit test. A non-significant value for the Hosmer–Lemeshow Chi-square test suggests an absence of biased fit. Analysis of the area under-the-receiver-operating-characteristic (ROC) curve (AUC) was constructed to assess the predictive strength. The nonparametric method of Delong was used to compare significant difference between AUCs. Sensitivity, specificity, and positive and negative likelihood ratios and predictive values were calculated at different cut-off values. Optimal cut-off points to maximize both sensitivity and specificity were also determined. Differences with *p*-values < 0.05 were considered to be statistically significant.

## 3. Results

### 3.1. Patient Characteristics

Of the total 707 NF patients, 40 (5.66%) died in the hospital. Patients who were discharged to home were considered to have a favorable outcome. The demographic and clinical characteristics and laboratory findings on the ED arrival are compared between survivors and non-survivors in Table 1. The level of serum albumin upon ED arrival in non-survivors was significantly lower than in survivors (*p* < 0.001). SOFA (sequential organ failure assessment) scores in non-survivors were significantly higher than in survivors, and were calculated during the first 24 h after admission. In the analysis of initial variables recorded at ED, non-survivors had higher proportion of acidosis (16.04% vs. 25.0%, *p* < 0.001), coagulopathy (14.24% vs. 27.5%, *p* < 0.001), higher blood lactate (2.8 vs. 6.6 mmol/L, *p* < 0.001), serum creatinine (1.7 vs. 2.4 mg/dL, *p* < 0.01), and CRP levels (124.1 vs. 161.7 mg/dL, *p* < 0.01). Age was similar in both groups.

### 3.2. Comparison of Data in NF Patients with Different Levels of Serum Albumin

Serum albumin was detectable with a range of 0.8–6.3 g/dL in 707 samples. The median serum albumin level measured upon ED arrival was 2.9 g/dL. A comparison of the demographic, clinical characteristics, and laboratory findings collected upon ED arrival and SOFA score among NF patients with different levels of serum albumin is shown in Table 2. The incidence of in-hospital mortality was significantly associated with decreased serum albumin levels (*p* < 0.001). A significant increase in the SOFA scores (*p* < 0.001), incidence of acidosis, episodes of hypotension, thrombocytopenia, and CRP concentrations were associated with decreases in the serum albumin levels. In contrast, the serum albumin levels (*p* < 0.001) were significantly decreased with increases in the blood lactate levels.

### 3.3. Association of Serum Albumin Level with In-Hospital Mortality

Univariate binary and multivariate logistic regression analyses were performed to investigate whether the serum albumin level upon ED arrival was independently associated with in-hospital mortality (Table 3). Age, SOFA score, laboratory findings collected on the ED arrival, clinical conditions, and comorbidities that were potentially associated with in-hospital mortality were included in the analyses. The following factors were significantly associated with in-hospital mortality in the unadjusted binary logistic regression analysis: SOFA score, acidosis, coagulopathy, episode of hypotension, blood lactate, albumin, creatinine, CRP value, and comorbidity with chronic kidney disease. The odds for in-hospital mortality decreased by 14% for every 1 g/dL increase in serum albumin (odds ratio (OR) = 0.86; 95% confidence interval (CI), 0.83–0.89; *p* < 0.001). The association of serum albumin levels with in-hospital mortality remained significant after adjusting for age and SOFA score (OR = 0.89; 95% CI, 0.85–0.96; *p* < 0.001). Multivariate logistic regression analysis identified blood lactate (OR = 1.17; 95% CI, 1.07–1.29; *p* < 0.001), serum albumin (OR per 1 g/dL increase = 0.92; 95% CI, 0.88–0.96; *p* < 0.001), and SOFA score (OR per 1-point increase = 1.15; 95% CI, 1.11–1.20; *p* < 0.001) as factors that were most significantly associated with in-hospital mortality in NF. The Hosmer–Lemeshow goodness-of-fit test for the multivariate logistic regression model was not significant (*p* = 0.611), indicating that the model adequately fits the data. Furthermore, the association between serum albumin levels and in-hospital mortality remained significant after adjusting for SOFA score and serum albumin (OR = 0.95; 95% CI, 0.90–0.98; *p* < 0.001).

### 3.4. Ability of Serum Albumin Level to Predict In-Hospital Mortality

**Figure 1:**
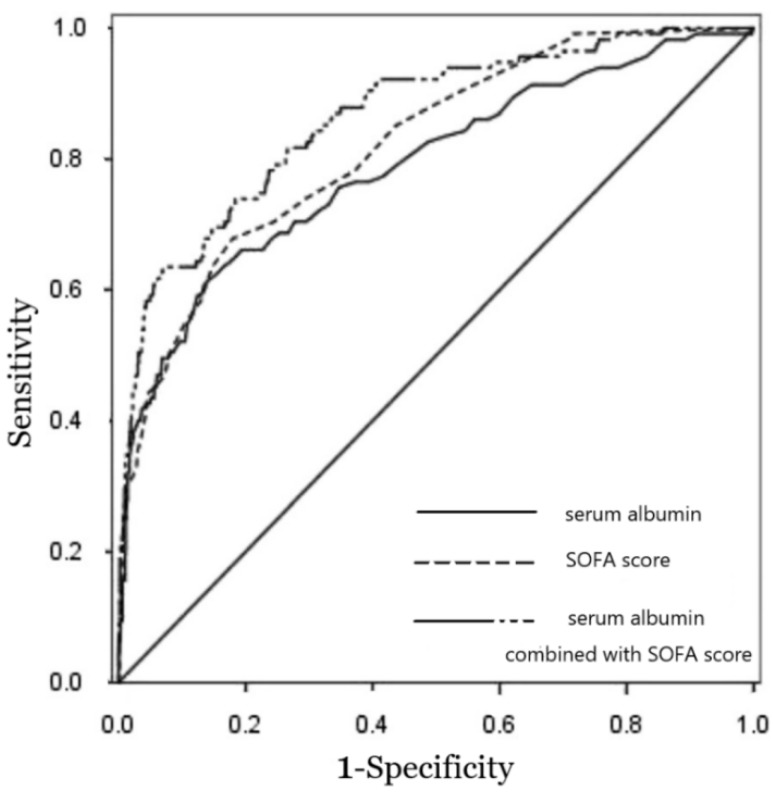
Receiver operating characteristic curves for the ability of serum albumin, sequential organ failure assessment (SOFA) score, and serum albumin combined with SOFA score to predict in-hospital mortality in necrotizing fasciitis (NF) patients (*n* = 707). The area under the receiver operating characteristic curve for serum albumin, SOFA score, and serum albumin combined with SOFA score were 0.77, 0.82, and 0.84, respectively, with a Hosmer–Lemeshow goodness-of-fit, *p* value > 0.05.

The predictive ability of serum albumin levels from NF patients upon ED arrival (*n* = 707) for in-hospital mortality (*n* = 40) was assessed. The level of serum albumin was predictive of in-hospital mortality and achieved AUC of 0.77 (95% CI, 0.72–0.82; *p* < 0.001). This AUC is similar to SOFA score (AUC = 0.82; 95% CI, 0.78–0.86; *p* < 0.001) for predicting the in-hospital mortality. The *p*-value for comparison of both AUCs was 0.324. Combining serum albumin levels with SOFA score improved the predictive performance (AUC = 0.84; 95% CI 0.76–0.92; *p* < 0.001), which is better than serum albumin alone (*p* = 0.013), but not significantly better than SOFA score alone (*p* = 0.274). Figure 1 shows the ROC curves and the AUC of the serum albumin level at ED arrival, SOFA score, and the combination of the serum albumin with SOFA score for predicting the in-hospital mortality of NF patients. Serum albumin displayed a sensitivity of 66% and a specificity of 74% at the optimal cut-off value of 3.2 g/dL. The positive and negative likelihood ratios were 2.25 and 0.48, respectively. SOFA score displayed a sensitivity of 67% and a specificity of 83% for predicting in-hospital mortality at the optimal cut-off score of 7.2, and the positive and negative likelihood ratios were 3.8 and 0.39, respectively. We also calculated the sensitivity and specificity of differing levels of serum albumin to predict in-hospital mortality in NF patients (Table 4). At the cut-off value of ≤4.0 of 96% and a specificity of 25% for predicting in-hospital mortality, the positive and negative likelihood ratios were 1.24 and 0.12, respectively. The specificity increased to 91%, and the positive likelihood ratio increased to 4.18 at the cut-off value of ≤2.5 g/dL, although the sensitivity decreased to 26%. The OR values of serum albumin at the levels above the set cut-off points are shown in Table 4.

## 4. Discussion

This study provides data on serum albumin levels and demonstrates that the serum albumin level upon ED arrival is significantly associated with in-hospital mortality in NF patients. A low serum albumin level at ED arrival is predictive of in-hospital mortality in NF patients. Some studies have demonstrated that albumin levels are associated with mortality in adults with NF [12,13,14,15]. To our knowledge, no studies verified the use of serum albumin as a prognostic index in NF patients who are admitted via ED. The observation that the extent of serum albumin levels is strongly linked with mortality indicates that serum albumin is a useful early predictor in identifying in-hospital mortality in NF patients. However, the use of a relatively high level of albumin (4.0 g/dL) was associated with high sensitivity (96%) for survival, and the use of a low level of albumin (2.5 g/dL) was associated with high specificity (91%) for mortality. These findings seem to have meaningful clinical significance. One contribution of this study is the use of SOFA score to control for the severity of the illness. Previous studies suggest that SOFA score is an important tool in predicting mortality and clinical outcomes in critically ill patients [16,17,18,19]. The association of ED serum albumin with in-hospital mortality in this study was independent of age and the severity of illness as assessed by SOFA score. The ROC curve analysis in the present study showed that the prognostic accuracy of serum albumin for in-hospital mortality (AUC = 0.77) was similar to that of SOFA score (AUC = 0.82). Because serum albumin at ED and SOFA score obtained within the first 24 h after admission are comparable in predicting mortality, we recommend assessing mortality risk with serum albumin at ED because it is simple to use. Some theories exist regarding the reasons that lower serum albumin levels, or hypoalbuminemia, may be associated with poor outcomes. Because synthesis and distribution of albumin may be directly associated with serum albumin level, factors that could affect albumin synthesis, distribution, or both need to be considered. Theoretically, decreased liver function or insufficient amino-acids intake may result in the hypoalbuminemia. In the present study, malnutrition was more frequently identified in the hypoalbuminemia group. A distribution disturbance between intravascular albumin levels and extravascular albumin levels may also exist. Certain conditions may decrease serum albumin levels such as pleural effusion, ascites, edema, or nephrosis. Nearly all drugs, including antibiotics, can bind with plasma protein and form protein–drug complexes. Because the unbound fraction of drugs exhibits a pharmacologic effect [20], lower serum protein levels would be beneficial. However, protein–drug complexes can escape via renal tubular secretion or hepatic metabolism [21] and may serve as a reservoir, resulting in a slow release of the drug in an active unbound form. These effects may be associated with an increased biological half-life of the drug; thus, contrary to the prior mechanism, higher serum protein levels would be beneficial in this case. In addition, it is not certain whether higher serum albumin levels are more beneficial or if a specific threshold of serum albumin level exists. Therefore, further investigations regarding this issue are needed. We cautiously suggest that the strategy in which serum albumin levels are maintained at the highest level possible may be beneficial based on this study. This finding implies that higher serum albumin level were associated with greater chance of survival. Based on this result, the authors thought that potential role of albumin administration would exist in NF. Although the study result was obtained using data at initial presentation and not after treatment, it could be representative of one point in the patients’ course of illness.

There were several limitations of this study. First, there was a temporal mismatch in evaluating mortality indicators. We compared the prognostic performance of an albumin value obtained on ED arrival with a SOFA score, which considers a range of values and includes the worst values obtained in the first 24 h of admission. The prognostic accuracy of the combination of serum albumin level and SOFA score was not significantly better than the use of SOFA score alone (*p* = 0.274), which might be explained by the discrepancy in the timescales for these two methods. Notably, the primary aim of the study was to evaluate the predictive value of serum albumin, when measured as a screening method at ED arrival, to predict mortality in patients with NF. Thus, serial changes in the albumin levels during the first 24 h of admission or post surgical intervention were not evaluated. Second, we could not collect long-term mortality data. Third, this was a retrospective observational single center study. Whether the results of the present study are replicable in the other regions is of question, because of potential differences in treatment quality and hospital resources. Further studies are needed to investigate the trends in the changes of albumin values and to explore whether the addition of the albumin value in the first 24 h to the SOFA score evaluation improves the prediction of mortality in NF patients.

## 5. Conclusions

Our study indicates that lower serum albumin levels upon ED arrival strongly predict in-hospital mortality in NF patients, even after adjusting for age, blood lactate, and SOFA score. However, the discrimination performance of a model using only albumin was not better than that of the SOFA score. These findings extend the knowledge of serum albumin as a clinical biomarker of mortality in critical illness.

## Figures and Tables

**Table 1:** Comparison of demographic and clinical characteristics and laboratory findings on emergency department (ED) arrival between survival and non-survival necrotizing fasciitis (NF) patients.

Characteristics	Survivors (*n* = 667)	Non-Survivors (*n* = 40)	*p*-Value
Age, years	57.2 (35.7–69.8) *	60.7 (39.3–82.6) *	0.09
SBP at triage, mmHg	146.5 (124.6–189.9) *	141.4 (104.6–198.5) *	0.93
DBP at triage, mmHg	85.1 (55.6–101.7) *	78.7 (44.1–99.5) *	0.79
SOFA score	4 (0–6) *	9 (5–23) *	<0.001
Episodes of hypotension, *n* (%)	86 (12.90%) ^#^	19 (47.50%) ^#^	<0.01
Hypothermia (BT < 36), *n* (%)	78 (11.70%) ^#^	10 (25.0%) ^#^	0.56
Hyperthermia (BT >= 38), *n* (%)	150 (22.49%) ^#^	9 (22.50%) ^#^	0.94
Acidosis, *n* (%)	106 (16.04%) ^#^	10 (25.0%) ^#^	<0.001
Coagulopathy, *n* (%)	95 (14.24%) ^#^	11 (27.5%) ^#^	<0.001
Thrombocytopenia, *n* (%)	69 (10.34%) ^#^	7 (17.5%) ^#^	0.23
Anemia, *n* (%)	88 (13.19%) ^#^	11 (27.50%) ^#^	0.08
Hypoxia, *n* (%)	65 (9.75%) ^#^	6 (15.0%) ^#^	0.07
Heart failure, *n* (%)	163 (24.43%) ^#^	11 (27.5%) ^#^	0.24
Diabetes mellitus, *n* (%)	193 (28.94%) ^#^	14 (35.0%) ^#^	0.26
Liver cirrhosis, *n* (%)	141 (21.14%) ^#^	13 (32.5%) ^#^	0.15
Chronic kidney disease, *n* (%)	207 (31.03%) ^#^	14 (35.05%) ^#^	0.34
Blood lactate (mmol/L)	2.8 (0.5–5.6) *	6.6 (1.2–11.8) *	<0.001
Serum albumin (g/dL)	3.1 (2.1–4.8) *	2.6 (1.9–3.6) *	<0.001
Serum creatinine (mg/dL)	1.7 (0.5–3.8) *	2.4 (0.9–6.6) *	<0.01
Serum glucose (mg/dL)	163 (121.5–188.9) *	192 (128.6–246.5) *	<0.01
CRP (mg/dL)	124.1 (56.1–174.5) *	161.7 (65.8–205.6) *	<0.01

* Values are median (interquartile range). ^#^ Numbers in parentheses denote percentages. BT, body temperature; CRP, C-reactive protein; DBP, diastolic blood pressure; SBP, systolic blood pressure; SOFA, sequential organ failure assessment.

**Table 2:** Comparison of demographic and clinical characteristics and laboratory findings on ED arrival among NF patients with different levels of serum albumin.

Admission Serum Albumin, g/dL	<2.5	2.5–3.0	3.0–3.5	3.5–4.0	>4.0	*p-*Value
*n*	95	286	187	86	53	
Age, years	60.1	57.6	54.8	52.3	51.1	<0.001
	(40.5–78.6) *	(42.3–70.8) *	(39.8–72.5) *	(43.7–78.1) *	(32.3–65.6) *	
SBP at triage, mmHg	141.2	137.5	132.9	133.7	132.5	0.58
	(121.4–167.9) *	(105.7–174.6) *	(111.2–164.7) *	(108.1–174.2) *	(109.6–178.5) *	
DBP at triage, mmHg	65.2	70.1	68.4	69.2	64.9	0.23
	(45.6–105.5) *	(55.8–101.6) *	(54.6–93.4) *	(49.5–94.8) *	(38.6–90.4) *	
Episode of hypotension, *n* (%)	25 (21.1%) ^#^	43 (18.5%) ^#^	32 (17.1%) ^#^	11 (12.6%) ^#^	6 (8.9%) ^#^	<0.001
SOFA score	8	7	6	4	3	<0.001
	(3–11) *	(2–8) *	(1–8) *	(0–7) *	(0–6) *	
Hypothermia (BT < 36), *n* (%)	12 (12.5%) ^#^	33 (15.4%) ^#^	26 (13.8%) ^#^	12 (14.2%) ^#^	8 (11.5%) ^#^	0.43
hyperthermia (BT ≥ 38), *n* (%)	27 (28.6%) ^#^	73 (25.6%) ^#^	42 (22.3%) ^#^	21 (24.9%) ^#^	11 (20.5%) ^#^	0.56
Acidosis, *n* (%)	34 (35.6%) ^#^	59 (20.5%) ^#^	33 (17.8%) ^#^	13 (15.6%) ^#^	3 (6.5%) ^#^	<0.001
Coagulopathy, *n* (%)	26 (27.1%) ^#^	61 (21.3%) ^#^	39 (20.6%) ^#^	16 (18.4%) ^#^	10 (18.3%) ^#^	0.12
Thrombocytopenia, *n* (%)	38 (40.3%) ^#^	93 (32.6%) ^#^	34 (18.3%) ^#^	14 (15.9%) ^#^	3 (6.3%) ^#^	<0.001
Anemia, *n* (%)	24 (25.4%) ^#^	61 (21.2%) ^#^	37 (19.8%) ^#^	14 (16.8%) ^#^	8 (15.5%) ^#^	0.45
Hypoxia, *n* (%)	14 (15.2%) ^#^	35 (12.4%) ^#^	26 (13.9%) ^#^	9 (10.7%) ^#^	5 (9.5%) ^#^	0.28
In-hospital mortality, *n* (%)	17 (17.9%) ^#^	11 (3.8%) ^#^	7 (3.7%) ^#^	3 (3.5%) ^#^	2 (3.8%) ^#^	<0.001
Diabetes mellitus, *n* (%)	32 (34.1%) ^#^	84 (29.7%) ^#^	58 (31.2%) ^#^	24 (28.3%) ^#^	12 (23.3%) ^#^	0.06
Liver cirrhosis, *n* (%)	39 (41.5%) ^#^	95 (33.2%) ^#^	57 (30.8%) ^#^	22 (25.4%) ^#^	6 (11.3%) ^#^	<0.001
Heart failure, *n* (%)	17 (18.6%) ^#^	61 (21.3%) ^#^	29 (15.4%) ^#^	15 (17.8%) ^#^	6 (10.9%) ^#^	0.49
Chronic kidney disease, *n* (%)	32 (33.8%) ^#^	75 (26.1%) ^#^	46 (24.9%) ^#^	16 (18.4%) ^#^	5 (9.5%) ^#^	0.23
blood lactate (mmol/L)	4.1	3.8	3.6	3.2	2.7	<0.001
	(2.9–5.6) *	(2.6–5.1) *	(2.3–4.5) *	(2.4–4.1) *	(1.5–3.9) *	
serum creatinine (mg/dL)	2.4	2.2	2.2	1.8	1.6	0.08
	(1.5–3.6) *	(1.6–3.3) *	(1.2–2.9) *	(1.3–2.8) *	(0.8–2.7) *	
serum glucose (mg/dL)	174	166	168	149	147	0.54
	(124–298) *	(126–254) *	(117–249) *	(115–224) *	(107–226) *	
serum CRP (mg/dL)	174	158	142	129	78	<0.001
	(56–225) *	(39–203) *	(31–195) *	(28–174) *	(25–151) *	

* Values are median (interquartile range). ^#^ Numbers in parentheses denote percentages. BT, body temperature; CRP, C-reactive protein; DBP, diastolic blood pressure; SBP, systolic blood pressure; SOFA, sequential organ failure assessment.

**Table 3:** Univariate and multivariate logistic regression analyses of variables potentially associated with in-hospital mortality in NF.

	Univariate Binary Logistic Regression		Multivariate Logistic Regression	
	OR (95% CI)	*p*-Value	OR (95% CI)	*p*-Value
Age	1.02 (0.96–1.08)	0.542	1.06 (0.96–1.16)	0.249
SOFA score	1.28 (1.06–1.51)	<0.001	1.15 (1.11–1.20)	<0.001 ^d^
Episode of hypotension	1.03 (1.01–1.05)	0.001	1.11 (0.63–1.97)	0.715
Episode of SpO_2_ <90%	1.58 (1.07–2.33)	0.325	1.14 (1.02–1.18)	0.459
Acidosis	1.05 (1.01–1.08)	<0.001	1.03 (0.94–1.12)	0.217
Coagulopathy	1.01 (1.00–1.01)	0.001	1.02 (1.01–1.08)	0.428
Thrombocytopenia	1.03 (0.98–1.08)	0.556	1.01 (0.93–1.03)	0.829
Anemia	1.05 (1.02–1.10)	0.065	0.99 (0.98–1.01)	0.913
Hypothermia	1.01 (1.00–1.01)	0.213	1.03 (0.98–1.07)	0.541
Hyperthermia	1.12 (1.02–1.15)	0.968	1.02 (1.01–1.12)	0.280
Blood lactate	1.35 (1.30–1.46)	<0.001	1.17 (1.07–1.29)	<0.001
Serum glucose	1.09 (1.01–1.23)	<0.01	1.94 (0.76–4.75)	0.580
Serum albumin	0.86 (0.83–0.89)	<0.001 ^a^	0.92 (0.88–0.96)	<0.001 ^b,c^
Serum CRP	1.11 (1.08–1.13)	<0.001	1.24 (1.18–3.27)	0.086
Serum creatinine	1.01 (1.00–1.01)	<0.001	1.12 (0.89–2.35)	0.306
Diabetes mellitus	1.05 (1.02–1.06)	0.133	1.18 (1.02–1.29)	0.102
Liver cirrhosis	1.02 (1.00–1.03)	0.506	1.05 (1.01–1.09)	0.020
Chronic kidney disease	1.02 (1.00–1.02)	<0.01	1.14 (1.05–1.18)	0.061

The *p*-value of the Hosmer–Lemeshow goodness-of-fit test for the multivariate logistic regression model was 0.611. ^a^ The association of serum albumin with in-hospital mortality remained significant after adjustment for age and SOFA score (OR = 0.89; 95% CI, 0.85–0.96; *p* < 0.001). ^b^ The association of the serum albumin level with in-hospital mortality remained significant after adjustment for blood lactate and SOFA score (OR = 0.95; 95% CI, 0.90–0.98; *p* < 0.001). ^c^ Odds ratio per 1 g/dL increase in albumin level. ^d^ Odds ratio per 1-point increase in SOFA score. CI, confidence interval; CRP, C-reactive protein; DBP, diastolic blood pressure; OR, odds ratio; SBP, systolic blood pressure; SOFA, sequential organ failure assessment.

**Table 4:** Odds ratio, sensitivity, and specificity for ED serum albumin at different levels to predict in-hospital mortality of NF.

ED Albumin, g/dL	OR ^a^ (95% CI)	*p*-Value	Sensitivity	Specificity	LR+	LR−	PV+	PV−
≤2.5	8.32 (4.46–16.35)	<0.001	26%	91%	4.18	0.76	0.56	0.83
≤3.0	4.88 (3.45–8.62)	<0.001	45%	76%	2.14	0.68	0.35	0.85
≤3.5	3.02 (1.95–8.39)	<0.001	79%	48%	1.61	0.37	0.29	0.91
≤4.0	1.98 (1.33–6.52)	<0.001	96%	25%	1.24	0.12	0.21	0.98

CI, confidence interval; LR+, likelihood ratio positive; LR−, likelihood ratio negative; OR, odds ratio; PV+, positive predictive value; PV−, negative predictive value. ^a^ Odds ratios of serum albumin at the levels above the set cut-off points.

## Availability of supporting data

Please contact author for data requests.

## Abbreviations

AUC	area under the receiver-operating-characteristic curve
CI	confidence interval
CKD	chronic kidney disease
CRP	C-reactive protein
DBP	diastolic blood pressure
ED	emergency department
LR+	likelihood ratio positive; LR−, likelihood ratio negative
NF	necrotizing fasciitis
OR	odds ratio
PV+	positive predictive value; PV−, negative predictive value
SBP	systolic blood pressure
SOFA	sequential organ failure assessment
